# Integrative proteomics and m6A microarray analyses of the signatures induced by METTL3 reveals prognostically significant in gastric cancer by affecting cellular metabolism

**DOI:** 10.3389/fonc.2022.996329

**Published:** 2022-11-16

**Authors:** Guisen Peng, Shuran Chen, Ni Zheng, Yuan Tang, Xu Su, Jing Wang, Rui Dong, Di Wu, Mingjie Hu, Yunli Zhao, Mulin Liu, Huazhang Wu

**Affiliations:** ^1^ School of Life Science, Anhui Province Key Laboratory of Translational Cancer Research, Bengbu Medical College, Bengbu, China; ^2^ Department of Gastrointestinal Surgery, Anhui Province Key Laboratory of Translational Cancer Research, First Affiliated Hospital of Bengbu Medical College, Bengbu, China; ^3^ School of Public Health, Bengbu Medical College, Bengbu, China

**Keywords:** METTL3, gastric cancer, m6A, oxidative phosphorylation, prognosis

## Abstract

METTL3-mediated RNA N6-methyladenosine (m6A) is the most prevalent modification that participates in tumor initiation and progression *via* governing the expression of their target genes in cancers. However, its role in tumor cell metabolism remains poorly characterized. In this study, m6A microarray and quantitative proteomics were employed to explore the potential effect and mechanism of METTL3 on the metabolism in GC cells. Our results showed that METTL3 induced significant alterations in the protein and m6A modification profile in GC cells. Gene Ontology (GO) enrichment indicated that down-regulated proteins were significantly enriched in intracellular mitochondrial oxidative phosphorylation (OXPHOS). Moreover, the protein-protein Interaction (PPI) network analysis found that these differentially expressed proteins were significantly associated with OXPHOS. A prognostic model was subsequently constructed based on the Cancer Genome Atlas (TCGA) and the Gene Expression Omnibus (GEO) databases, and the high-risk group exhibited a worse prognosis in GC patients. Meanwhile, Gene Set Enrichment Analysis (GSEA) demonstrated significant enrichment in the energy metabolism signaling pathway. Then, combined with the results of the m6A microarray analysis, the intersection molecules of DEPs and differential methylation genes (DMGs) were significantly correlated with the molecules of OXPHOS. Besides, there were significant differences in prognosis and GSEA enrichment between the two clusters of GC patients classified according to the consensus clustering algorithm. Finally, highly expressed and highly methylated molecules regulated by METTL3 were analyzed and three (AVEN, DAZAP2, DNAJB1) genes were identified to be significantly associated with poor prognosis in GC patients. These results signified that METTL3-regulated DEPs in GC cells were significantly associated with OXPHOS. After combined with m6A microarray analysis, the results suggested that these proteins might be implicated in cell energy metabolism through m6A modifications thus influencing the prognosis of GC patients. Overall, our study revealed that METTL3 is involved in cell metabolism through an m6A-dependent mechanism in GC cells, and indicated a potential biomarker for prognostic prediction in GC.

## Introduction

Gastric cancer is a common global disease, ranking fifth in incidence and fourth in mortality among malignant tumors (1). Its onset and progression are influenced by several factors, including epigenetic, genetic, and environmental factors (2). In recent years, there has been an increase in the study of epigenetic processes, owing to the gradual accumulation of epigenetic alterations, resulting in gain-of-function of oncogenes and loss-of-function of tumor suppressor genes (3), both of which have been shown to play a key role in gastric tumorigenesis (4, 5). Therefore, it is particularly to further explore the roles and underlying mechanism of epigenetic modification in tumorigenesis and development.

N6-methyladenosine (m6A) is the most prevalent mRNA epigenetic modification in eukaryotes which regulated by methyltransferase complexes (“writers”), demethylases (“erasers”) and RNA-binding proteins (“readers”) (6, 7). m6A mRNA modifications have presently garnered attention because of their crucial roles in mediating gene expression and determining cell fate. Numerous studies have established that m6A can regulate the occurrence and development of GC. METTL3 is an m6A-modified core methyltransferase (8, 9), it can effectively promote the proliferation, invasive and migratory abilities, and epithelial-mesenchymal transition (10, 11) of GC cells; conversely, its knockdown reduces lipid accumulation and cell viability (12). m6A-seq analysis has shown that MYC target genes are mediated by METTL3 through an m6A modification manner (13). Meanwhile, m6A can positively regulate glycolysis in cancer cells by regulating pyruvate dehydrogenase kinase 4 (PDK4) (14), a crucial factor that directs carbon flux from OXPHOS to glycolysis. However, the involvement of METTL3 in the metabolism of GC cells and its possible underlying mechanisms warrants further investigations. Herein, proteomics, in conjunction with m6A microarray analysis were utilized to explore the role and potential mechanism of METTL3 in GC cell metabolism and further reveal the possible mechanism of its involvement in the occurrence and development of GC, thereby providing a theoretical basis for the treatment of GC.

## Material and methods

### Cell culture and establishment of a stable cell line overexpressing METTL3

The human GC cell line BGC-823 was purchased from the Institute of Cell Research of the Chinese Academy of Sciences in Shanghai. The BGC-823 cells were cultured in DMEM (Procell Life Science&Technology Co.,Ltd) supplemented with with 10% fetal bovine serum (FBS; Gibco) and 1% penicillin/streptomycin (HuaXiao gene Technology) in a 37°C, 5% CO_2_ incubator with a humidified atmosphere. Lentivirus-mediated METTL3-overexpressing and control were purchased from Jikai Gene Chemical Technology Co.,Ltd (Shanghai, China). Establishment and screening of METTL3-overexpressing stable cell models according to the instructions of Jikai Gene Technology Co., Ltd. Briefly, when the cells in the logarithmic growth phase, the control (NC group) and METTL3 overexpressing lentivirus were added in BGC-823 cells, puromycin (4 μg/mL) was used to screen the successfully transfected cells for the subsequent experiments. The western blotting analyses were performed as described previously (15), and the primary antibodies used in western blotting experiments are as follows: β-Actin (66009-1-Ig, Proteintech); AVEN (25846-1-AP, Proteintech); DNAJB1 (13174-1-AP, Proteintech); METTL3 (15073-1-AP, Proteintech).

### Quantitative proteomics by multiplexed tandem mass tag combined with mass spectrometry

Proteins were extracted from control and overexpressed METTL3-overexpressed BGC-823 cells, respectively. The tandem analysis of the high-precision mass spectrometer was performed by Novogene Technology Co., Ltd. (Beijing, China). The MS proteomics data have been deposited to the ProteomeXchange Consortium *via* the PRIDE partner repository with the dataset identifier PXD036773. The results of protein quantitation were statistically analyzed by T-test. The proteins whose expression levels significantly differed between experimental and control groups (p<0.05 and fold-change ≥ 1.2) were defined as differentiallyexpressed proteins (DEPs) (16).

### m6A RNA methylation quantification

The total RNA of the control and METTL3 overexpression group were extracted by Trizol for m6A-tagged mRNA quantification. Then, m6A epitranscriptomic microarrays were performed by Aksomics, Inc (Shanghai, China), and data were acquired using the Agilent feature extraction software (version 11.0.1.1). We have uploaded the RAW data to the public repository Gene Expression Omnibus (GSE213499). Screening criteria for multiple variations and statistically significant (P-value) thresholds were used to identify differences between the two m6A-methylated RNA groups. Stratified clustering was performed to determine differences in m6A methylation patterns. For the analysis of the results of m6A methylation microarray, the fold-change ≥ 1.2, P< 0.05 was defined as DMGs (17).

### Raw data acquisition and processing

The gene expression profiles of 804 GC patients were downloaded from the GSE84437 dataset of the TCGA and GEO (http://www.ncbi.nlm.nih.gov/geo/) databases, and the “limma” package in R was used to correct the data. Mutation data from GC patients is processed by a Perl script. The gene expression profile of a total of 804 patients in TCGA and GEO were both in FPKM format and log2 normalized. To further explore the biological significance of proteins and genes, we used the hypergeometric distribution of “clusterprofiler” package to analyze and visualize data for enrichment analysis of functions and pathways. In GO and Kyoto Encyclopedia of Genes and Genomes (KEGG) analysis, the adjust P< 0.05 and FDR q< 0.25 are considered significant. The gene sets of oxidative phosphorylation, carbohydrate metabolism, amino acid metabolism and lipid metabolism were obtained from MSigDB (https://www.gsea-msigdb.org/gsea/msigdb/). Z-score is calculated using the “GOplot” package.

### Construction of the protein–protein interaction network

Protein-protein interactionPPI analysis of METTL3 was performed using STRING (https://string-db.org/) database. In addition, we use Cytoscape was applied (version3.8.2) to visualize the relationship between DEPs.

### Consensus clustering

To explore the function of molecules regulated by METTL3, GC patients were c-ategorized into various clusters using the “ConsensusClusterPlus” package (50 iteratio-ns, 80% resampling rate Pearson correlation, https://www.bioconductor.org/packages/release/bioc/html/ConsensusClusterPlus.html).

### Establishment of the model based on METTL3-related genes

Univariate and multivariate cox proportional risk analyses were conducted using the “survival” and “survminer” packages. The criterion of P < 0.05 was selected as the filtering threshold. The Least Absolute Shrinkage and Selection Operator (LASSO) was conducted to avoid over-fitting and to delete those tightly correlated genes. Tenfold cross-validation was employed to select the minimal penalty term (λ). The formula of the risk score was constructed as follows:


$$\text{Risk score }=\sum_{i=1}^{n}Coef_{i}*x_{i}$$

where Coef*_i_* represents the coefficients and X*_i_* represents the normalized count of each hub genes. The median risk assessment is achieved using “ggrisk” in R. Risk models were used to divide GC patients into high- and low-risk groups based on median risk scores. The “ggplot2” and “survival” packages were used to plot Kaplan-Meier (K-M) survival curves for the overall survival (OS) of different GC subtypes. The time-dependent subject work characteristic (ROC) curve and the area under the ROC curve (AUC) were used to assess the sensitivity and specificity of the model. The AUC value was calculated by the “survival ROC” packages. The “pRRophetic” package was used to predict clinical drug therapy for the different GC subtypes.

## Results

### Down-regulated proteins were significantly associated with oxidative phosphorylation in METTL3 overexpressing GC cells

**Figure 1:**
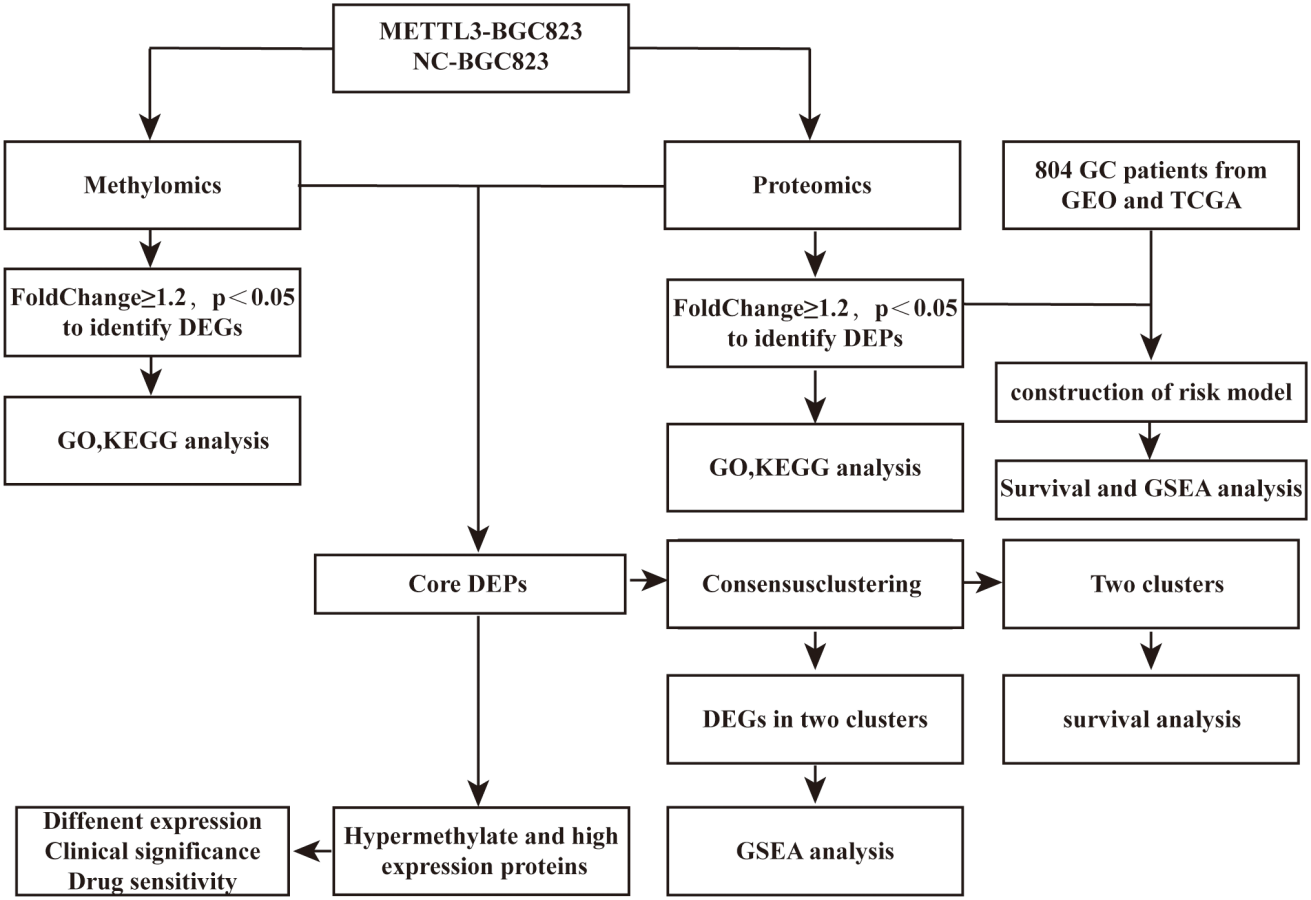
Flowchart depicting the process of this study. DEPs, differentially expressed proteins; DMGs, differential methylation genes; DEGs, differentially expressed genes.

**Figure 2:**
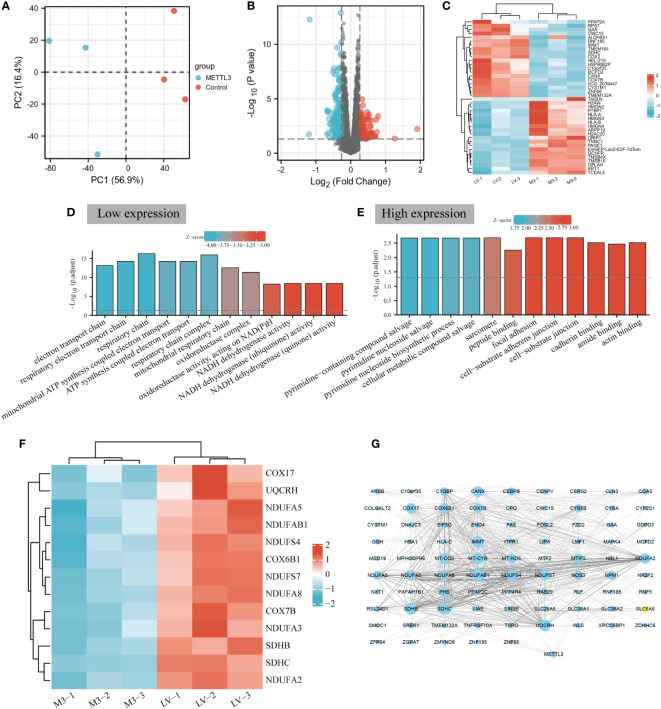
METTL3 is involved in cellular energy metabolism in BGC-823 cells. **(A)** Principal component analysis of METTL3 overexpressd and control samples in BGC-823 cells. **(B)** Volcano plot analysis of differentially expressed proteins regulated by METTL3, the red and blue dots represent upregulated and downregulated proteins, respectively, and black dots represent no difference. **(C)** Heatmap of the top 20 most significantly up-regulation (red) and down-regulation (blue) DEPs. **(D)** GO enrichment analysis of downregulated proteins. The “GOplot” R packet was used to calculate Z-score; a positive Z-score indicated that it may be positive adjustment, while a negative Z-score indicated that it may be a negative adjustment. The larger the absolute value, the higher the degree of regulation. **(E)** GO enrichment analysis of up-regulated proteins. **(F)** Heatmap of the number of DEPs and OXPHOS intersecting molecules expressed in GC cells. **(G)** PPI network diagram of METTL3 and low-expressed DEPs. The larger the area of the dots, the more number of connections between the corresponding nodes and other nodes, the stronger the co-expression relationship.

To investigate the global protein expression profiles in response to overexpression METTL3, mass spectrometry-based proteomic experiments with TMT labeling were performed by Novogene Biotech Co., Ltd (Beijing, China) to explore the comprehensive changes in proteomic profiles in BGC-823 cells (The efficiency of METTL3 overexpression was shown in **Supplementary Figure 1**). The flowchart of the study is as illutrated in **Figure 1**. Principal component analysis (PCA) determined that the METTL3 overexpression and control samples were independently clustered and exhibited significant differences between the two groups (**Figure 2A**). Based on this, a volcano plot was constructed for visualization of DEPs between the two groups, and 173 differerntially expressed proteins were identified (fold-change ≥1.2, P<0.05, 79 up-regulated and 95 down-regulated proteins) following METTL3 overexpression (**Figure 2B**). Then, the top 20 with up- and down-regulated DEPs were selected to construct a heat map for visual analysis (**Figure 2C**). GO is one of the most extensively used tools for classifying biological entities into functionally related groups, and the GO results demonstrated that the downregulated proteins regulated by METTL3 overexpression were mainly enriched in cellular energy metabolism processes, such as ATP metabolism process, mitochondrial dehydrogenase complex, mitochondrial respiratory chain, and NADH dehydrogenase activity especially in intracellular OXPHOS (**Figure 2D**, **Supplementary Table 1**). Correspondingly, upregulated proteins were mainly enriched in the pyrimidine nucleoside biosynthetic process, cellular metabolic compound salvage, cadherin binding and other biological processes (**Figure 2E**, **Supplementary Table 2**). To explore the role of METTL3-regulated DEPs in OXPHOS, the 173 DEPs were intersected with the gene sets of the OXPHOS pathway obtained from GSEA, and a total of 13 intersecting molecules were screened to generated a heatmap to visualize their expression in GC cells (**Figure 2F**). Interestingly, these proteins were all significantly downregulated in METTL3-overexpressed GC cells compared to the control group. Finally, a PPI network diagram was constructed by using Cytoscape (version 3.8.2), and a strong correlation was identified between METTL3 and low-expressed DEPs (**Figure 2G**). Taken together, these results indicated that the down-regulated proteins regulated by METTL3 are involved in cellular energy metabolism, especially in mediating mitochondria-related oxidative phosphorylation.

### METTL3-regulated DEPs affects the prognosis of GC patients and are involved in energy metabolism pathways

**Figure 3:**
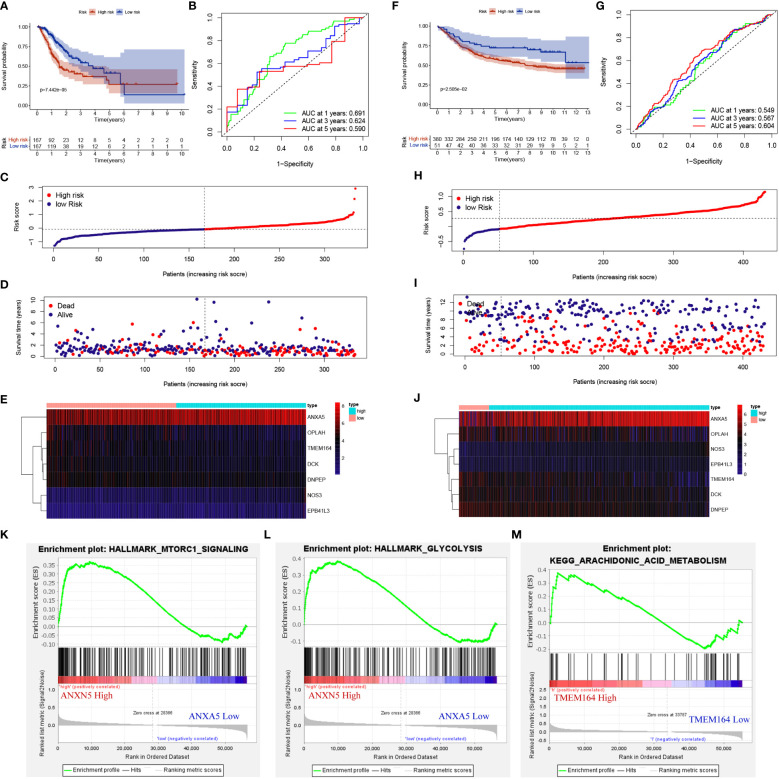
METTL3-regulated DEPs affected the prognosis of GC patients and were involved in cellular energy metabolism. **(A, F)** K-M survival curves for high- and low-risk groups in training set and testing set. **(B, G)** ROC curves for sensitivity and specificity of risk models used to predict 1-, 3- and 5- year survival in training set and testing set. **(C, D, H, I)** Scatter plot of DEPs risk scores and patient survival, with red and blue dots representing high- and low-risk patients in training set and testing set. **(E, J)** Expression of gene used to construct risk models in training set and testing set. GSEA gene enrichment of high-risk group with ANXA5 **(K, L)** and TMEM164 **(M)** high expression.

To evaluate the impact of METTL3-regulated DEPs on the prognosis of GC patients, firstly, LASSO regression and multivariate Cox analysis were performed on 173 DEPs to determine the best candidate DEPs, and 7 (DCK, TMEM164, ANXA5, OPLAH, NOS3, DNPEP, EPB41L3) independent prognostic DEPs were identified for building risk models, and the coefficients for each DEP in the risk model were -0.03, -0.02, 0.00, -0.01, 0.09, -0.01 and 0.04 respectively (**Supplementary Table 3**). Risk model was constructed based on the TCGA dataset), and the GSE84437 dataset (as the validation set) to verify the model values. The K-M survival curve showed that the overall survival (OS) of high-risk patients in the train group was significantly lower than that in the low-risk group (**Figure 3A**, **Supplementary Table 3**). The ROC curve showed AUC values of 0.691, 0.624 and 0.590 for the probability of survival at 1, 3 and 5 years for GC patients (**Figure 3B**). In addition, as the risk score increased, the number of patients who died increases significantly (**Figures 3C, D**), and we also generated a heatmap to visualize the differential expression of 7 risk factors in high- and low-risk patients (**Figure 3E**). The risk score also significantly distinguished the prognosis of patients in the external independent GEO dataset (**Figures 3F–J**). Finally, gene set enrichment analysis (GSEA) of the 7 DEPs-related to the high- and low-risk groups was conducted to explore the potential mechanisms affecting the prognosis of GC patients. Interestingly, the high-risk group with increased expression levels of ANXA5 and TMEM164 was significantly enriched in energy metabolism-related signaling pathways such as mTORC1, glycolysis, and arachidonic acid metabolism (**Figures 3K-M**). Collectively, these results indicated that METTL3-regulated DEPs might influence the prognosis of GC patients through energy metabolism-related signaling pathways.

### METTL3-mediated m6A modification involved in cellular oxidative phosphorylation

**Figure 4:**
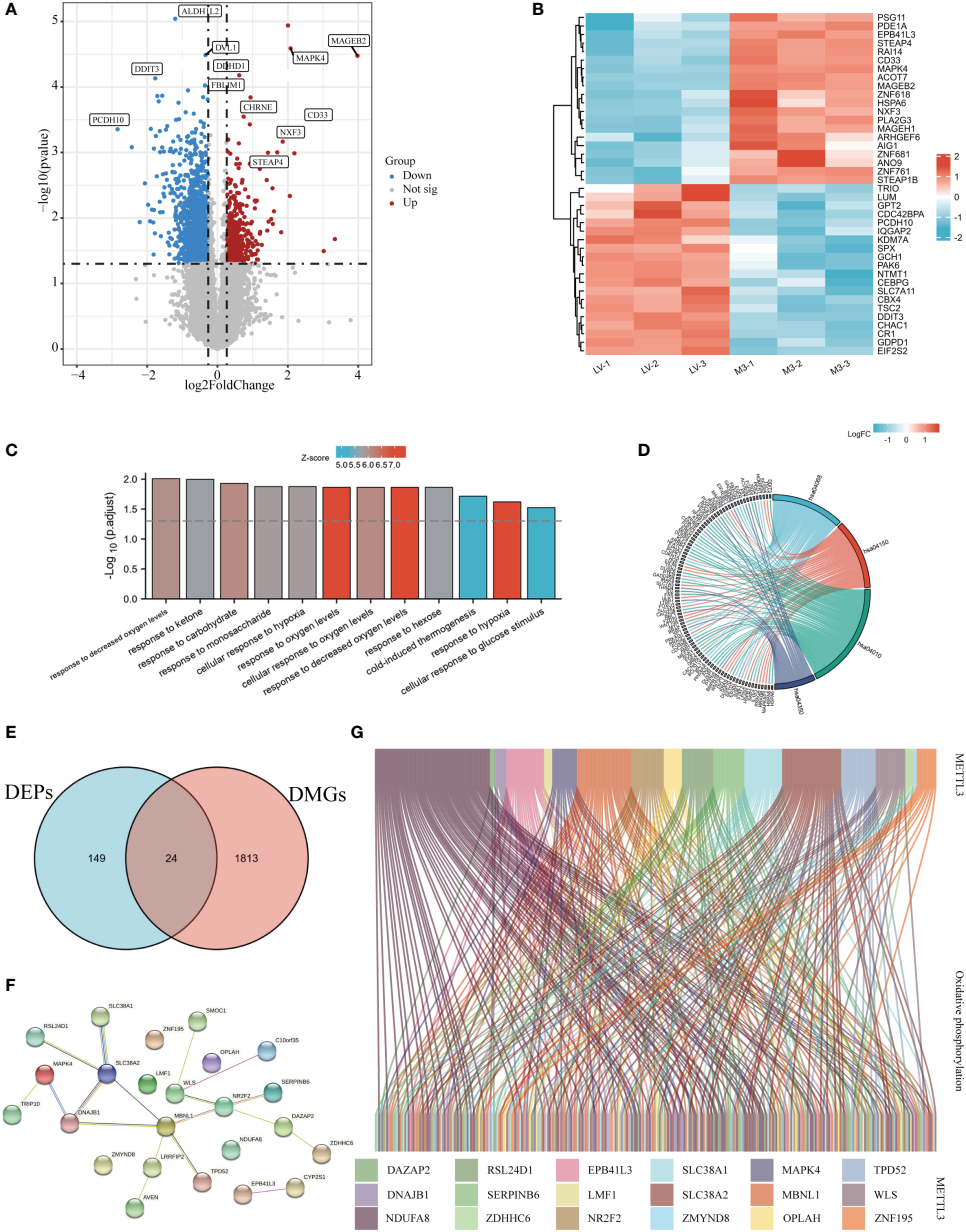
METTL3 was involved in oxidative phosphorylation through m6A regulation of target genes. **(A)** Volcano plot of METTL3-regulated DMGs, red and blue dots represented up- and down-regulated proteins, respectively, and gray dots represented no difference. **(B)** A heatmap-based visualization for the top 20 methylated genes significantly up- and down-regulated by METTL3; red and blue represent up- and down-regulated genes, respectively. GO **(C)** and KEGG **(D)** enrichment analyses of differentially expressed genes regulated by METTL3. **(E)** Venn diagram of the intersection of DEPs and DEGs identifying 24 molecules. **(F)** The PPI network was constructed based on the core DEPs. **(G)** Sankey diagram analysis of the correlation between core DEPs and oxidative phosphorylation molecules obtained from GSEA official website; genes are represented by different colors, and each connecting line represents an intermolecular connection with correlation coefficients > 0.3.

Previous studies have demonstrated that METTL3-mediated m6A methylation could regulate gene expression *via* post-transcriptional alterations (10). To explore the possible impact of m6A modification on the expression of METTL3-induced DEPs, m6A-mRNA & lncRNA epitranscriptomic microarrays were carried out in METTL3 overexpressing BGC-823 cells. The results showed that METTL3 overexpression resulted in a total of 1837 DMGs, with 430 up-regulated and 1407 down-regulated methylated genes (**Figure 4A**). Next, the top 20 methylated genes that were significantly up- and down-regulated were chosen to generate a heatmap (**Figure 4B**). The GO enrichment analysis showed that DMGs regulated by METTL3 were significantly enriched in pathways that responded to oxygen level, carbohydrates and the cellular response to glucose stimulation and so on (**Figure 4C**). KEGG analysis found that the DEGs were predominantly enriched in energy metabolism-related signaling pathways such as MAPK, FOXO, mTOR, and TGF-β (**Figure 4D**, **Supplementary Table 4**).

To further validated whether METTL3-mediated m6A methylation is involved in regulating the expression of genes related to energy metabolism, we took the intersection of DEPs and DMGs obtained from proteomic and m6A methylation analyses,and a total of 24 core DEPs were obtained for further analysis (**Figure 4E**). The PPI network constructed with core DEPs displayed the interactions between these core DEPs (containing 24 nodes and 33 edges, P<0.05, **Figure 4F**). Then, the relationship between core DEPs and oxidatively phosphorylated molecules was explored, Sankey diagram delineated the correlation between METTL3-regulated core DEPs and oxidatively phosphorylated molecules with correlation coefficients > 0.3 (**Figure 4G**). In short, these results suggest that METTL3-mediated m6A modification is involved in the oxidative phosphorylation pathway.

### Significant differences in metabolic pathways and prognostic characteristics of GC patients with different molecular subtypes

**Figure 5:**
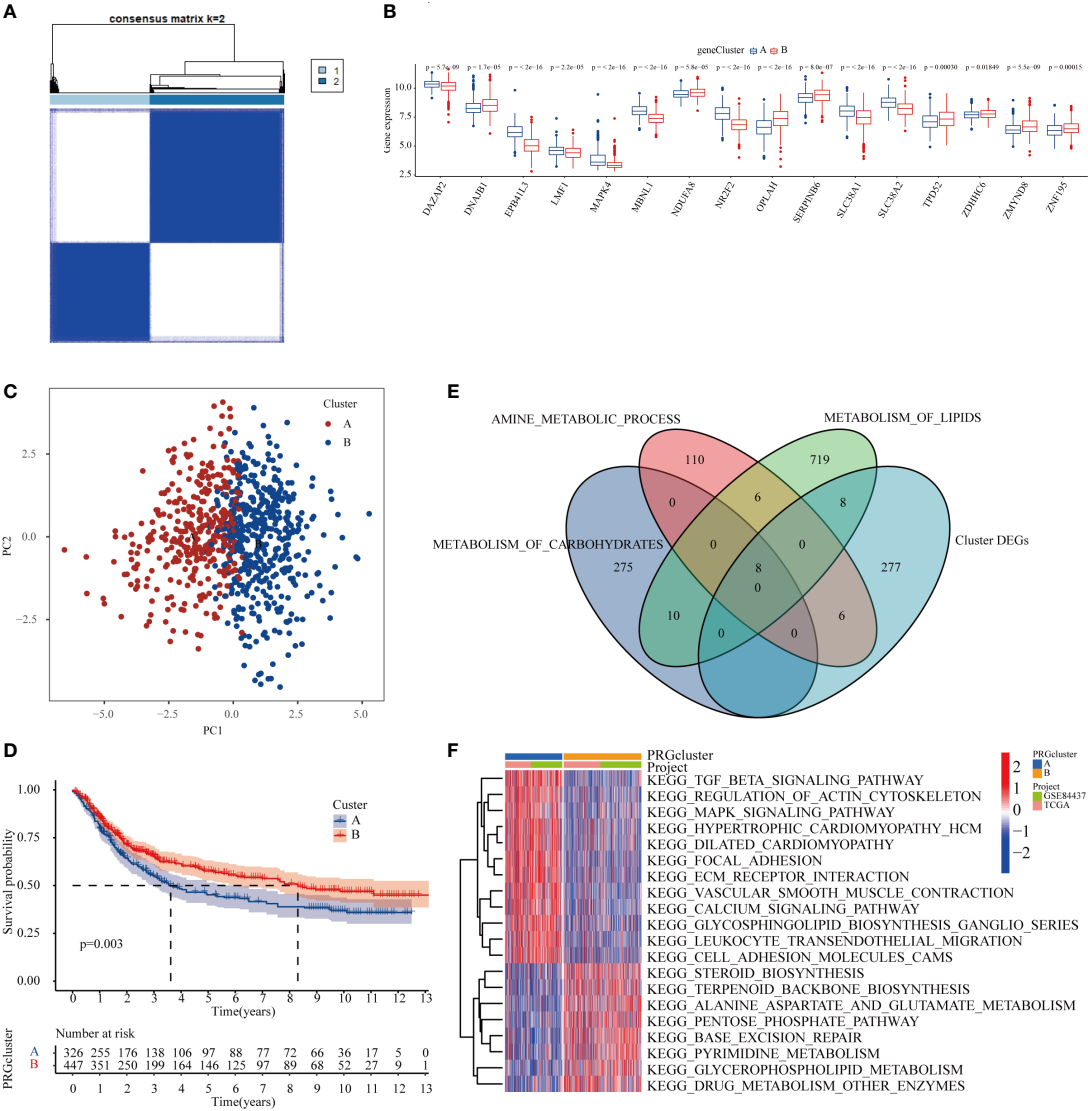
METTL3 participated in the metabolic process and affected the prognosis of GC patients by regulating the level of m6A modification and protein expression. **(A)** Consensus clustering of GC patients according to MTTL3-regulated m6A modification and protein expression based on TCGA and GEO datasets. **(B)** Histogram demostrating that the 16 genes were significantly differentially expressed between the two subtypes. **(C)** The PCA plot displayed that there was a significant difference between the two clusters of samples. **(D)** Kaplan-Meier survival curve showed that the OS of cluster B GC patients was significantly better than that of cluster **(A)**. **(E)** Intersection genes between the DEGs and three major energy metabolism pathways in patients with subtypes A and **(B)**. **(F)** Heatmap of GSEA enrichment analysis of DEGs in patients with subtypes A and B.

To further explore the effect of METTL3-regulated m6A modification and protein expression on cellular metabolism and its relationship with the prognosis of GC patients, the mRNA expression and profiling data were downloaded from the TCGA and GEO databases. A consensus clustering algorithm was used to divide GC patients into two subtypes according to the 24 core DEPs. To determine the optimal number of clusters, differences in 24 core DEPs expression clustering stability were assessed using the “Consensus Cluster Plus” package, and when the consensus matrix k=2, there was no crossover between the GC samples of cluster A and B (**Figure 5A**, **Supplementary Table 5**, **Supplementary Figure 1**). The histogram illustrated that the expression levels of 16 core DEPs (16 of 24 core DEPs above) were significantly different between the two subtypes (**Figure 5B**). Meanwhile, the PCA plot showed significant differences between the two clusters which reflected not only the specificity of the samples in clusters A and B, but also the classification accuracy of the samples (**Figure 5C**). Next, the correlation between the two subtypes and prognostic characteristics in GC patients was evaluated, and the result of the Kaplan-Meier survival curve showed that the OS of cluster B was significantly superior to that of cluster A in GC patients (**Figure 5D**). Finally, a Venn diagram was employed to visualize the relationship between DEGs in the two subtypes and the three major energy metabolism pathways including carbohydrate metabolism, amino acid metabolism, and lipid metabolism. The results showed that the DEGs overlapped with the three major energy metabolism pathways, and a total of 8 lipid metabolism-related, 8 carbon oxide metabolism-related and 6 amino acid metabolism-associated molecules appearing in the intersection (**Figure 5E**). GSEA enrichment analysis found that cluster A samples were significantly enriched in signaling pathways such as TGF-β and MAPK, whereas cluster B samples were significantly enriched in signaling pathways such as pentose phosphate pathway, pyrimidine metabolism, and drug metabolism (**Figure 5F**). Taken together, these results indicated that METTL3 might participate in metabolic processes and influence the prognosis of GC patients by mediating the level of m6A modification and protein expression, suggesting that these differentially expressed genes may serve as novel prognostic markers for GC patients.

### METTL3-mediated gene expression regulation is significantly associated with poor clinical prognosis and treatment of GC patients

**Figure 6:**
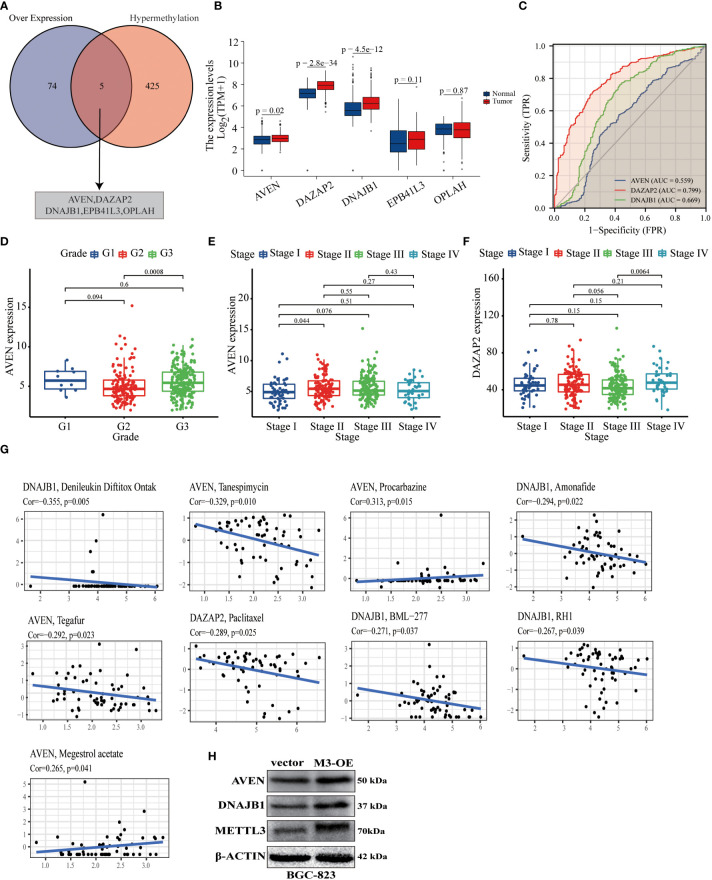
METTL3-mediated gene expression was associated with poor clinical prognosis and treatment of GC patients. **(A)** Venn diagram of the intersection of up-regulated DEPs and DMGs by proteomic and m6A epitranscriptomic microarray analysis; the intersected genes were AVEN, DAZAP2, DNAJB1, EPB41L3, OPLAH. **(B)** Differential expression of five intersecting molecules in GC and normal gastric mucosal tissue. **(C)** ROC curves of AVEN, DAZAP2 and DNAJB1 used to predict 5- year survival in GC based on datasets from the TCGA and GEO databases. **(D–F)** The expression levels of AVEN and DAZAP2 were correlated with the clinicopathological grade and stage of GC patients. **(G)** The expression levels of AVEN, DAZAP2 and DNAJB1 in GC were correlated with antitumor drugs, with and Cor representing the correlation coefficient. **(H)** The expression levels of AVEN and DNAJB1 were significantly upregulated by METTL3 overexpression in BGC-823 cells.

Previous studies have reported that METTL3-dependent m6A methylation leads to RNA hypermethylation and participates in the regulation of downstream biological processes (18). We combined the up-regulated DEPs obtained by proteomic analysis and the hypermethylated genes obtained by m6A epitranscriptomic microarray analysis in GC cells, and a total of 5 core highly expressed and hypermethylated molecules were identified (**Figure 6A**, **Supplementary Table 6**). Further comprehensive analysis based on TCGA data indicated that the expression level of 3 genes (AVEN, DAZAP2, DNAJB1) were significantly higher in GC tumor tissues than in normal tissues (**Figure 6B**). Next, to investigate whether AVEN, DAZAP2, and DNAJB1 could serve as potential prognostic biomarkers in GC patients by receiver operating characteristic (ROC) curves, the results showed that the AUC values of the three molecules were 0.559, 0.799, and 0.669, respectively, showing a predictive value for clinical prognosis of GC patients (**Figure 6C**). Notably, the boxplot results showed that the expression levels of AVEN and DAZAP2 were significantly correlated with clinicopathological grade and stage of GC patients (**Figure 6D-F**). The correlation between the expression of AVEN, DAZAP2, DNAJB1 and anticancer drugs in GC patients was analyzed, and the results showed that AVEN, DAZAP2, and DNAJB1 were significantly correlated with antitumor drugs (tanspiromycin, procarbazine, amonafide, megestrol acetate, etc.) (**Figure 6G**). Finally, AVEN and DNAJB1 (19, 20), which have DNA damage and tumor formation roles in tumors were selected to verify their protein expression levels in BGC-823 control and METTL3 overexpression cell lines, and the results showed that the AVEN and DNAJB1 level were significantly upregulated by METTL3 expression (**Figure 6H**). Collectively, the above results show that the high degree of m6A modification and increased protein expression mediated by METTL3 are significantly correlated with the prognosis and antitumor drugs of GC patients, signifying that they are promising novel biomarkers and potential clinical therapeutic targets for GC.

## Discussion

N6-methyladenosine is the prominent dynamic mRNA modification which was governed by methyltransferase complex (“writers”), demethylases (“erasers”), and RNA-binding proteins (“readers”) (7). Emerging evidence shows that m6A modification is associated with malignant phenotypes of tumors such as cell proliferation, differentiation, invasion and metastasis and functions as an oncogene in the development and progression of cancers (21–24). Similarly, the increased expression of METTL3 involved in regulating cell excessive proliferation, multidrug resistance, and distant metastasis in GC (25–27). Recent studies have shown that increased expression of METTL3 was associated with tumor cell glycolysis metabolism and sensitivity to glycolytic stress in hepatocellular carcinoma (28), and N(6)-methyladenosine regulates the glycolysis of cancer cells through PDK4 (14). Likewise, METTL3 can promote the glycolysis of GC cells through HDG-activated GLUT4 and ENO2, thereby promoting tumor progression (29), and can also attenuate oxidative stress and apoptosis in by regulating the Keap1/Nrf2 pathway (30), implying that METTL3 can regulate cellular oxidative phosphorylation in GC cells. The above studies signaled that METTL3 may play a decisive role in metabolic reprogramming in carcinogenesis. However, the biological functions and underlying mechanisms of METTL3 in the metabolic reprogramming of GC have not yet been fully elucidated.

High-throughput omics technologies, including genomics, transcriptomics, epigenomics, proteomics, etc., have been widely used in biological research (31–33). Herein, proteomics in conjunction with m6A methylome was used to analyze the potential role and mechanism of METTL3 in GC cell metabolism. Our results found significant alterations in the protein and m6A modification profile induced by METTL3 overexpression in BGC-823 cells. Furthermore, GO and KEGG analyses revealed showed that the differentially expressed proteins were predominantly enriched in oxidative phosphorylation-related pathways. Earlier studies have shown that proteomics outperforms transcriptomics in risk stratification across tumor types (34). Therefore, the risk of GC patients was divided into two groups according to the differentially expressed proteins screened by proteomics. GC patients were categorized as either high- or low-risk based on the 7 differentially expressed proteins regulated by METTL3, and the K-M survival curve delineated that there was a significant difference in the prognosis between the two groups. In addition, univariate and multivariate Cox analyses illustrated that models constructed with differentially expressed proteins possessed higher accuracy in predicting patient prognosis than the commonly used clinical TNM staging. It is worthwhile pointing out that GSEA enrichment analysis determined that the differentially expressed ANXA5 and NR2F2 were involved in the hypoxia, glucose metabolism and mTOR signaling pathways. It has been reported that administration of ANXA5 following cisplatin therapy restored the immunosuppressive effects of the tumor microenvironment (35). Furthermore, prior studies have also established that ANXA5 participates in the regulation of oxidative stress processes in testicular cells (36, 37). A recent study demonstrated that Wnt/NR2F2/GPX4 promoted acquired chemoresistance by suppressing ferroptosis with high consumption of GSH, and also revealed the key role of NR2F2 in the regulation of tumor metabolism (38). Numerous studies have revealed the regulatory effects of ANXA5 and NR2F2 on metabolism. Combined with the proteomics results, we speculate that ANXA5 and NR2F2 may be crucial targets of METTL3 for regulating oxidative phosphorylation, albeit further functional studies are necessitated to validate this hypothesis.

In view of the important m6A methylation modification function of METTL3, the differentially methylated genes and proteins caused by METTL3 were intersected, and a total of 24 molecules were screened for further analyses. These molecules can effectively separate GC patients into two groups with different prognoses, and the TGF-β and MAPK signaling pathways were significantly activated in GC patients with worse prognoses. At the same time, the differential genes between the two types were also involved in metabolic pathways such as glucose metabolism, lipid metabolism and amino acid metabolism.

Since considering that m6A modification can promote protein expression by stabilizing mRNAs (36, 39), we further screened highly expressed and hypermethylated molecules which regulated by METTL3, and the AVEN, DAZAP2, DNAJB1, EPB41L3, and OPLAH molecules were selected for further analysis The results showed that the expression levels of AVEN and DAZAP2 were significantly correlated with the clinicopathological grade and stage of GC patients. The former has been shown to exert anti-apoptotic and DNA damage regulation effects, whereas the latter is also involved in modulating DNA damage in tumor cells (19, 40, 41). Nevertheless, whether METTL3 exerts biological functions through these genes must be further verified by *in vitro* and *in vivo* experiments.

METTL3 is an important m6A methylation modification gene facilitating tumor progression by promoting tumor proliferation, migration, angiogenesis and other pathways (42). Nevertheless, the regulation of METTL3 on tumor cell metabolism, especially oxidative phosphorylation remains unknown. Therefore, this study combined proteomics and methylomes to explore their effect on GC cell metabolism, especially oxidative phosphorylation, and we anticipate that our study will provide an advanced understanding of METTL3 and cell metabolism.

## Data availability statement

The original contributions presented in the study are included in the article/**Supplementary Material**. Further inquiries can be directed to the corresponding authors.

## Author contributions

Conception and design: HW, ML, YZ. Data analysis and interpretation: SC, GP, NZ, YT. Manuscript writing: HW, GP, SC, NZ, YT, XS, JW, RD, YZ, MH, DW. All authors contributed to the article and approved the submitted version.

## Funding

This work was supported by the Anhui Natural Science Foundation (1908085MH257, 2108085MH291), the Natural Science Foundation for Colleges and Universities of Anhui Province (KJ2021A0737, KJ2021A0787, KJ2021ZD0092), the 512 Talent Cultivation Plan of Bengbu Medical College (by51201107).

## Conflict of interest

The authors declare that the research was conducted in the absence of any commercial or financial relationships that could be construed as a potential conflict of interest.

## Publisher’s note

All claims expressed in this article are solely those of the authors and do not necessarily represent those of their affiliated organizations, or those of the publisher, the editors and the reviewers. Any product that may be evaluated in this article, or claim that may be made by its manufacturer, is not guaranteed or endorsed by the publisher.
