# Supplementary material for: Integrative proteomics and m6A microarray analyses of the signatures induced by METTL3 reveals prognostically significant in gastric cancer by affecting cellular metabolism
Source: Front Oncol. 2022 Nov 16;12:996329. doi: 10.3389/fonc.2022.996329 (PMC9709115; doi:10.3389/fonc.2022.996329)
Supplement: Supplementary file 1 [file DataSheet_1.docx]

## Supplementary Figure 1

**
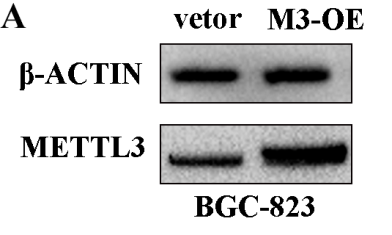
**

**Supplementary Figure 1** (A) Expression levels of β-ACTIN and METTL3 proteins in BGC-823 control cells and METTL3-overexpressing cells

## Supplementary Figure 2

##
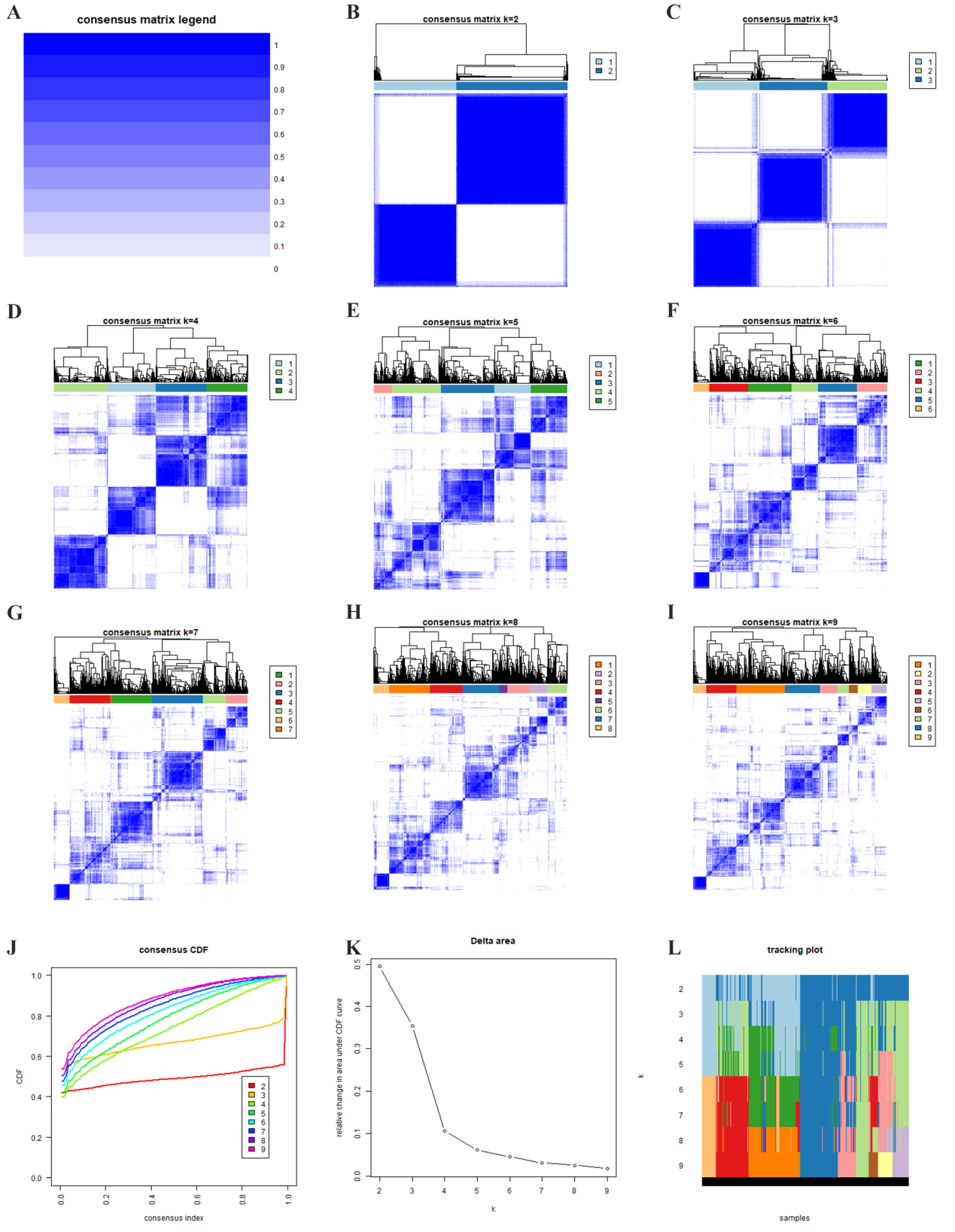


**Supplementary Figure 2** (A-L) The plot of consensus CDF and relative change in area under CDF curve (B) when the consensus matrix k=2, there was no crossover between the GC samples of cluster A and B.
